# Neuropeptide deficient mice have attenuated nociceptive, vascular, and inflammatory changes in a tibia fracture model of complex regional pain syndrome

**DOI:** 10.1186/1744-8069-8-85

**Published:** 2012-11-28

**Authors:** Tian-Zhi Guo, Tzuping Wei, Xiaoyou Shi, Wen-Wu Li, Saiyun Hou, Liping Wang, Kazutake Tsujikawa, Kenner C Rice, Kejun Cheng, David J Clark, Wade S Kingery

**Affiliations:** 1 Physical Medicine and Rehabilitation Service, Veterans Affairs Palo Alto Health Care System, Palo Alto, CA, USA; 2 Anesthesiology Service, Veterans Affairs Palo Alto Health Care System, Palo Alto, CA, USA; 3 Department of Anesthesiolgy, Stanford University School of Medicine, Stanford, CA, USA; 4 Department of Immunology, Graduate School of Pharmaceutical Sciences, Osaka University, Osaka, Japan; 5 Drug Design and Synthesis Section, Chemical Biology Research Branch, National Institute on Drug Abuse and National Institute on Alcohol Abuse and Alcoholism, NIH, Bethesda, MD, USA

**Keywords:** Substance P, Calcitonin gene-related peptide, Fracture, Complex regional pain syndrome, Inflammation, Pain, Cytokine, Nerve growth factor

## Abstract

**Background:**

Distal limb fracture in man can induce a complex regional pain syndrome (CRPS) with pain, warmth, edema, and cutaneous inflammation. In the present study substance P (SP, Tac1^−/−^) and CGRP receptor (RAMP1^−/−^) deficient mice were used to investigate the contribution of neuropeptide signaling to CRPS-like changes in a tibia fracture mouse model. Wildtype, Tac1^−/−^, and RAMP1^−/−^ mice underwent tibia fracture and casting for 3 weeks, then the cast was removed and hindpaw mechanical allodynia, unweighting, warmth, and edema were tested over time. Hindpaw skin was collected at 3 weeks post-fracture for immunoassay and femurs were collected for micro-CT analysis.

**Results:**

Wildtype mice developed hindpaw allodynia, unweighting, warmth, and edema at 3 weeks post-fracture, but in the Tac1^−/−^ fracture mice allodynia and unweighting were attenuated and there was no warmth and edema. RAMP1^−/−^ fracture mice had a similar presentation, except there was no reduction in hindpaw edema. Hindpaw skin TNFα, IL-1β, IL-6 and NGF levels were up-regulated in wildtype fracture mice at 3 weeks post-fracture, but in the Tac1^−/−^ and RAMP1^−/−^ fracture mice only IL-6 was increased. The epidermal keratinocytes were the cellular source for these inflammatory mediators. An IL-6 receptor antagonist partially reversed post-fracture pain behaviors in wildtype mice.

**Conclusions:**

In conclusion, both SP and CGRP are critical neuropeptide mediators for the pain behaviors, vascular abnormalities, and up-regulated innate immune responses observed in the fracture hindlimb. We postulate that the residual pain behaviors observed in the Tac1^−/−^ and RAMP1^−/−^ fracture mice are attributable to the increased IL-6 levels observed in the hindpaw skin after fracture.

## Background

Complex regional pain syndrome (CRPS) usually develops after a regional injury and presents with distal limb nociceptive, vascular, and bone changes that exceed the expected clinical course of the inciting injury in both magnitude and duration, frequently resulting in significant motor impairment and disability. CRPS symptoms usually gradually improve over the first year after injury, but persistent CRPS is a serious problem, resulting in edema, pain, weakness, contractures, and bone loss. Over 80% of chronic CRPS patients are severely disabled [1]. The debate regarding the underlying mechanisms of CRPS has been dynamic and controversial, and despite extensive investigation, the pathophysiology of this condition remains undefined and there is considerable disagreement on whether any treatment for CRPS is effective [2-5]. There is clearly a need for mechanism based effective and safe treatments for this debilitating complication of injury.

Early CRPS clinical findings include vasodilatation, redness, warmth, increased spontaneous protein extravasation, edema, periarticular bone loss, pain, and allodynia in the injured limb [6]. Population-based studies indicate that distal limb fracture is the most common cause of CRPS [7,8] and we have developed and characterized a rat fracture CRPS model mimicking the clinical condition. After distal tibia fracture and cast immobilization for 4 weeks the rats develop chronic unilateral hindlimb warmth, edema, facilitated spontaneous protein extravasation, periarticular osteoporosis, hindlimb unweighting, and allodynia, changes paralleling those observed in CRPS patients [9]. The vascular and nociceptive changes characteristic of the early stages of CRPS suggest an inflammatory process, yet there is little evidence for an immunocyte response in these patients [10-15]. Similarly, in the rat CRPS model we observe no evidence of macrophage, T-cell, or neutrophil infiltration in the hindpaw skin at 4 weeks post-fracture [16]. On the other hand, there is compelling evidence that neurogenic inflammatory responses are exaggerated in the CRPS limb and in the rat fracture model.

Neurogenic inflammation is mediated by the depolarization of small sensory afferents in the skin, triggering the release of substance P (SP) and calcitonin gene-related peptide (CGRP), neurotransmitters that activate their receptors in the dermal vasculature to induce protein extravasation and vasodilatation. Electrically evoked extravasation and vasodilatation responses are enhanced in CRPS patients and when SP is microdialyzed in the skin there is exaggerated protein extravasation, direct evidence of facilitated neurogenic inflammatory responses in CRPS [17,18]. Furthermore, serum levels of SP and CGRP are elevated in CRPS patients [19-21]. Similarly, after tibia fracture in rats there is increased SP and CGRP expression in the sciatic nerve and in serum, up-regulated SP NK1 receptor expression in the endothelial cells and keratinocytes of the hindpaw skin, and SP evoked extravasation and edema responses are enhanced in the fracture hindlimb [22]. Treating fracture rats with an SP NK1 receptor antagonist reduced hindlimb allodynia, warmth, and edema, suggesting that exaggerated SP signaling contributes to the development of CRPS-like changes after fracture [9].

Inflammatory cytokine levels are up-regulated in CRPS skin [23-25], and levels of interleukin 1b (IL-1b), interleukin 6 (IL-6), tumor necrosis factor a (TNFa), and nerve growth factor (NGF) are elevated in the hindpaw skin keratinocytes of fracture rats [16,26-28]. Treating fracture rats with the TNF inhibitor etanercept, the IL-1 receptor antagonist anakinra, or the anti-NGF antibody tanezumab reduced hindpaw allodynia and unweighting at 4 weeks post-fracture [26,27,29].

Collectively, these data support the hypothesis that fracture causes exaggerated SP and CGRP signaling in the skin, resulting in increased extravasation, edema, warmth, and pain behavior. The aims of current study were to develop a tibia fracture CRPS model in mice and utilize this model in SP deficient (Tac1^−/−^) and in CGRP receptor (RAMP1^−/−^) deficient mice to directly test the hypothesis that neuropeptide signaling is required for the development of the post-fracture innate immunity responses contributing to the development of pain behavior in the fracture model.

## Results

### Characterizing the WT, Tac1^−/−^, and RAMP1^−/−^ mice

**Figure 1:**
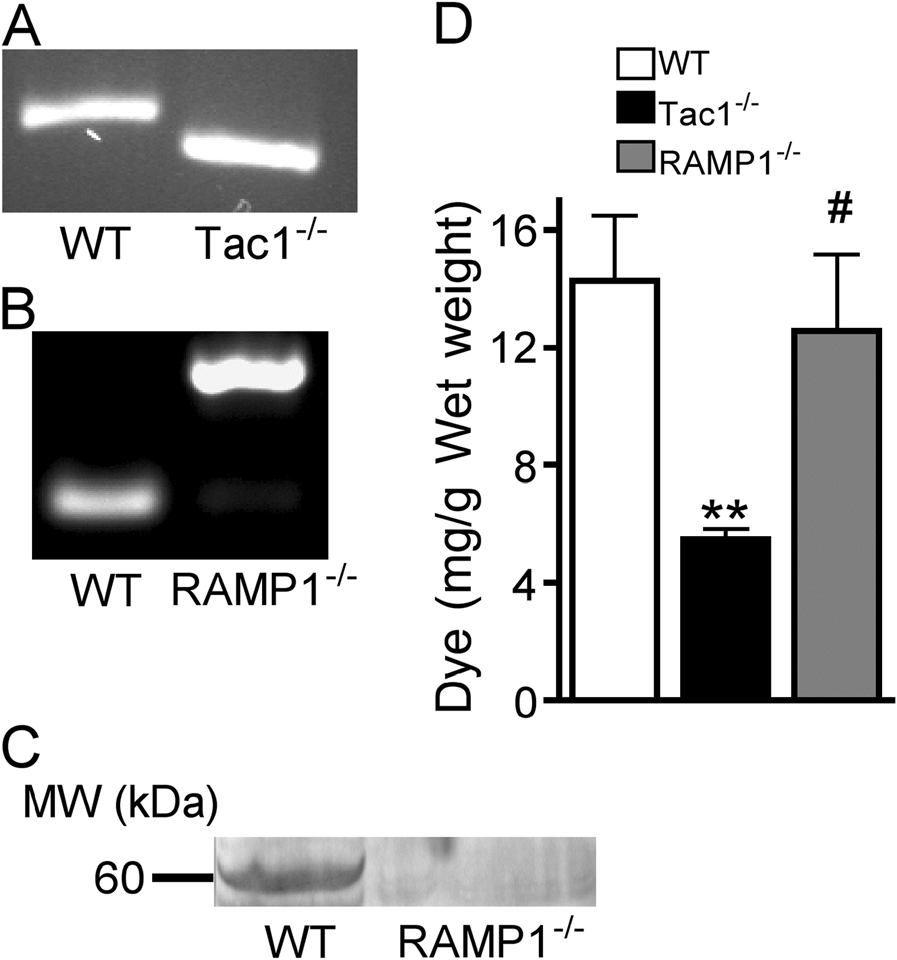
**Genotyping and phenotyping of substance P (SP) deficient (Tac1**^**−/−**^**) and CGRP receptor deficient (RAMP1**^**−/−**^**) mice.** PCR products of tail DNA from wildtype (WT) and Tac1^−/−^ mice yielded 190 and 170-bp PCR fragments, respectively (**A**). The knockout mutant PCR fragment was shorter due to the targeted deletion. WT and mutant RAMP1^−/−^ alleles yielded 108 and 240-bp PCR fragments, respectively. Western blot assay was used to demonstrate that RAMP1 protein expression in the hindpaw skin was abolished due to disruption in the genomic structure of the RAMP1 allele (**C**) Electrically evoked extravasation responses were quantified by measuring Evans blue dye content in the hindpaw skin after 5 minutes of sciatic stimulation at C-fiber intensity (**D**). Tac1^−/−^ mice extravasation responses were reduced 60% vs WT mice, but no decrease in extravasation was observed in the RAMP1^−/−^ mice (n = 6 per cohort). These results indicate that neuronal SP release can induce microvascular protein extravasation into the cutaneous interstitial space, but neuronal CGRP release is an ineffective mediator of cutaneous extravasation. **P <0.01 *vs* WT, #P < 0.05 *vs* Tac1^−/−^.

In the intact unfractured mice there were no differences between wildtype (WT), SP deficient (Tac1^−/−^), and CGRP receptor deficient (RAMP1^−/−^) mice in terms of hindpaw von Frey thresholds, weight bearing, hindpaw temperature, or hindpaw thickness. Figure 1A illustrates that the Tac1^−/−^ mice yielded shorter PCR product (size 130 bp) on agarose gel, whereas WT mice yielded longer product (180 bp). WT and RAMP1^−/−^ mice yielded PCR products of 108 and 240 bp, respectively (Figure 1B). Western blot assay demonstrated a protein band at 60.5 kDa in WT mice that was absent in the RAMP1^−/−^ mice, confirming knockout of RAMP1 protein in transgenic mice (Figure 1C). To confirm functional inhibition of SP signaling in the Tac1^−/−^ mice, the sciatic nerve was exposed and electrically stimulated for 5 minutes at C-fiber intensities and Evans blue dye extravasation was quantified in the hindpaw skin of WT, Tac1^−/−^ and RAMP1^−/−^ mice. Electrically evoked extravasation was reduced by 62% in the SP deficient mice (*vs* WT controls, p < 0.01), but no loss of extravasation response was observed in the RAMP1^−/−^ mice (n = 6 per cohort, Figure 1D).

### Nociceptive and vascular changes after fracture

**Figure 2:**
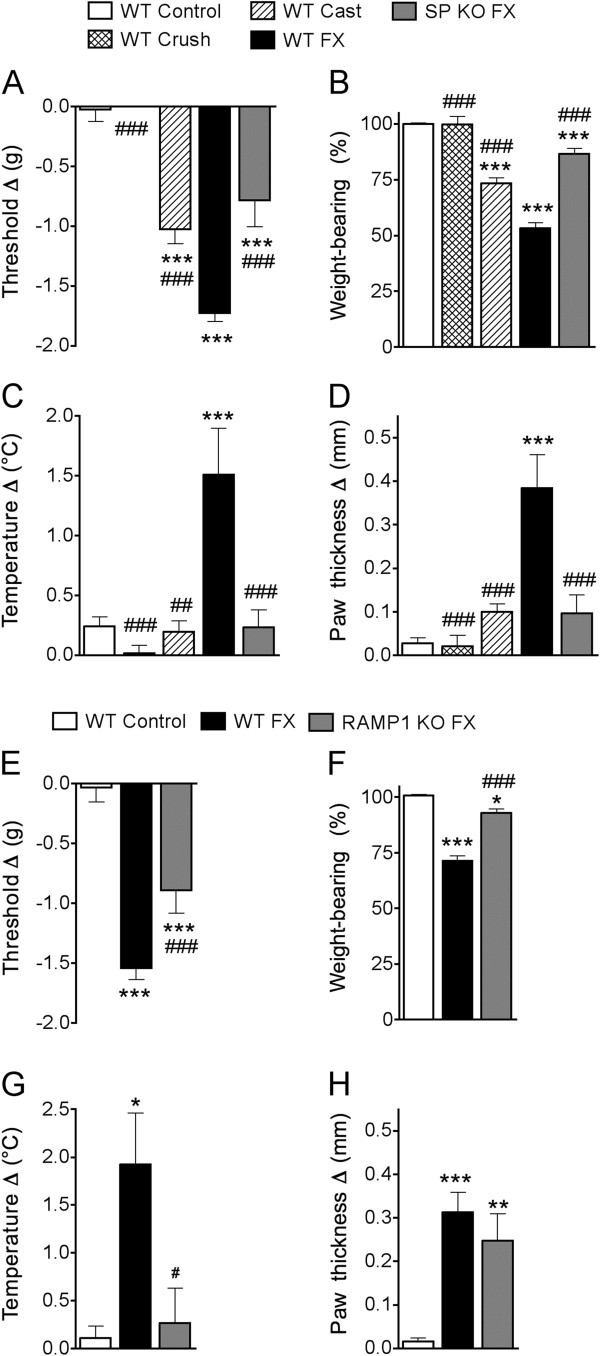
**Wildtype (WT), substance P deficient (SP KO, Tac1**^**-/-**^**), and CGRP receptor deficient (RAMP1 KO, RAMP1**^**-/-**^**) mice underwent behavioral testing and then distal tibia fracture (FX) with hindlimb casting for 3 weeks, then the cast was removed and behavioral testing repeated (n = 10-12).** Baselines did not differ between WT, SP KO, and RAMP1 KO mice. Controls included WT mice with no treatment (WT Control), WT mice with crush injury (no FX) of the distal ankle (WT Crush), and WT mice with hindlimb casting (no FX) for 3 weeks (WT Cast). WT Crush mice failed to develop nociceptive or vascular changes, but WT Cast mice developed hindpaw von Frey allodynia and unweighting, but no warmth or edema (**A**-**D**). The absence of SP signaling (SP KO FX) partially inhibited post-FX allodynia (**A**) and unweighting (**B**) and completely blocked warmth (**C**) and edema (**D**), compared to the WT FX mice. Similarly, the loss of CGRP signal (RAMP1 KO FX) partially inhibited post-FX allodynia (**E**) and unweighting (**F**), completely blocked hindpaw warmth (**G**), and had no effect on hindpaw edema (**H**), compared to WT fracture mice. For detailed descriptions of the hindpaw behavioral assays see *Methods*. A negative von Frey threshold value (**A**, **E**) represents mechanical allodynia on the fracture side. A value less than 100% for weight bearing represents unweighting (**B**, **F**). Positive values for temperature (**C**,**G**) or thickness (**D**,**H**) represent warmth or edema. * P < 0.05, **P < 0.01, and ***P < 0.001 vs WT Control, #P < 0.05, and ##P < 0.01 vs WT FX.

After baseline testing, 3 month-old male WT, Tac1^−/−^, and RAMP1^−/−^ mice (n = 10–12 per cohort) underwent a right distal tibia fracture with 3 weeks cast immobilization. Two additional groups of control mice were used in these experiments, WT mice that had a crush injury (no fracture) to the distal tibia using the same hemostat used to fracture the tibia (WT Crush, n = 8) and WT mice that underwent hindlimb cast immobilization for 3 weeks with no fracture (WT Cast, n = 8). Figures 2A-E show that the WT crush mice failed to develop any nociceptive or vascular changes, but the 3 week cast immobilized control mice (WT Cast, no fracture) did develop hindpaw von Frey allodynia and unweighting, but not warmth and edema. Figure 2 illustrates that at 3 weeks post-fracture most of the WT mice (n = 40) developed hindpaw allodynia (89.3% incidence, inclusion based on a minimum reduction of at least 1.3 g in the von Frey hindpaw withdrawal threshold, fracture *vs* contralateral side), hindpaw unweighting (85% incidence, based on at least 30% unweighting of the hindpaw, fracture *vs* contralateral side), hindpaw warmth (80% incidence, based on a minimum reduction in hindpaw skin temperature of 0.5°C, fracture *vs* contralateral hindpaw), and hindpaw edema (87.5% incidence, based on a minimum increase in hindpaw thickness of 0.1 mm, fracture *vs* contralateral side).

Figures 2A and E illustrate that after cast removal both the WT and the transgenic mice had developed reduced withdrawal thresholds to von Frey testing, but the Tac1^−/−^ and RAMP1^−/−^ mice had significantly less allodynia than WT fracture mice. Similarly, at 3 weeks post-fracture both the WT and the transgenic mice unweighted the injured hindlimb, but both the Tac1^−/−^ and RAMP1^−/−^ mice had significantly less hindpaw unweighting than the WT mice (Figures 2B,F).

The WT mice also exhibited increased hindpaw temperature and thickness at 3 weeks post-fracture, *vs* the contralateral paw, but the SP deficient mice failed to develop significant hindpaw warmth or edema (Figures 2C,D). The RAMP1^−/−^ fracture mice failed to develop hindpaw warmth after fracture, but had a similar degree of hindpaw edema as the WT fracture mice (Figures 2G,H).

**Figure 3:**
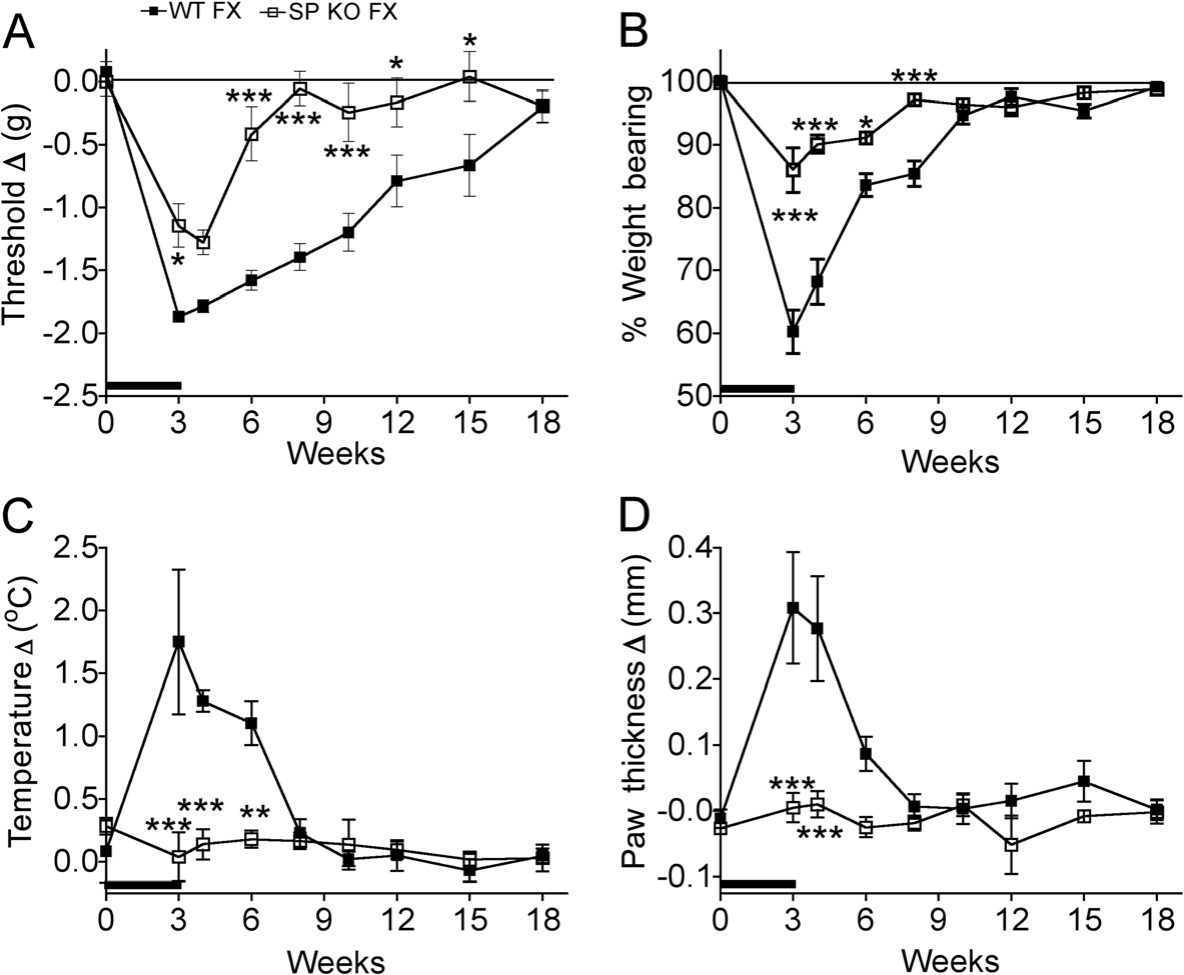
**After baseline testing, WT and SP KO (Tac1**^**−/−**^**) mice underwent a right distal tibia fracture (FX) and the hindlimb was casted for 3 weeks (black bar on x-axis), then the cast was removed and the animals were retested at 3, 4, 6, 8, 10, 12, 15, and 18 weeks post fracture (n = 7 per cohort).** The methods for testing and calculating the graph values are described in Figure 2. (**A**) Hindpaw allodynia was present at 3 weeks in both groups and resolved by 18 weeks post-fracture in the WT mice and by 6 weeks post-fracture in the SP KO cohort. When compared at the same post-fracture time points the magnitude of allodynia was consistently less in the SP KO mice than that observed in the WT mice. (**B**) Hindpaw unweighting was present at 3 weeks after fracture in both groups and resolved by 10 weeks in the WT mice and by 8 weeks the SP KO mice, but the magnitude of unweighting was consistently less in the SP KO mice than the WT. Hindpaw warmth (**C**) and edema (**D**) were present at 3 weeks post-fracture in the WT mice and resolved by 8 weeks post-fracture for warmth and 6 weeks for edema. Hindpaw warmth and edema was not observed in the SP KO mice at any time point. *P < 0.05, **P < 0.01, and ***P < 0.001 *vs* WT FX mice at the same time point.

Figure 3 illustrates the time course for the resolution of hindlimb allodynia, unweighting, warmth, and edema after tibia fracture in WT and SP deficient mice. Hindpaw von Frey allodynia resolved by 18 weeks post-fracture in WT mice and by 6 weeks after fracture in the Tac1^−/−^ mice (Figure 3A). Hindlimb unweighting recovered by 10 weeks post-fracture in the WT mice and by 8 weeks after fracture in the Tac1^−/−^ mice (Figure 3B). Figure 3C illustrates that temperature in the injured hindpaw remained significant significantly elevated for 6 weeks after fracture in the WT mice, but there was no temperature increase at any time point in the SP deficient mice. Hindpaw edema resolved by 6 weeks post-fracture in the WT mice and no edema was observed at any time point in the Tac1^−/−^ mice (Figure 3D). There were no contralateral effects of fracture on hindpaw temperature or thickness at any time point in either mouse strain.

### Effects of fracture on hindpaw skin cytokine and NGF levels

**Figure 4:**
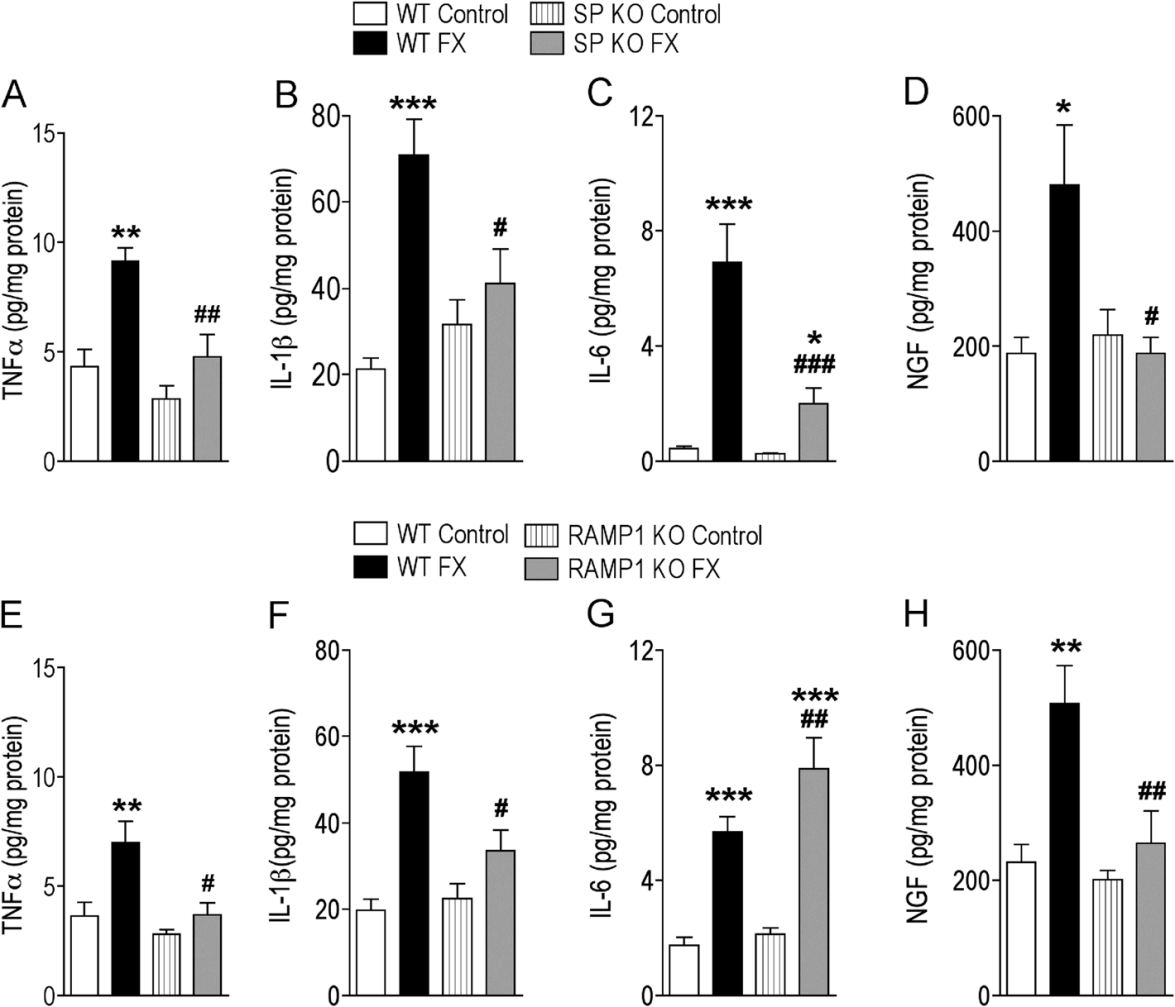
**Determination of hindpaw skin cytokine and NGF levels at 3 weeks after fracture (FX) in WT, SP deficient (SP KO, Tac1**^**−/−**^**), and CGRP receptor deficient (RAMP1 KO, RAMP1**^**−/−**^**) mice. TNFα (A, E), IL-1β (B, F), IL-6 (C, G) and NGF (D, H) production in hindpaw skin were determined by Bio-Rad bead suspension array (for cytokines) and EIA (for NGF).** Baseline cytokine and NGF levels were the same in all 3 groups of mice. Tibia fracture induced a significant increase in all three cytokine levels and NGF production in WT FX group *vs* WT Controls, however, fracture did not induce increased TNFα (**A**), IL-1β (**B**), or NGF (**D**) levels in SP KO FX mice compared to unfractured SP KO Control mice. The IL-6 (**C**) levels were elevated in the SP KO FX mice, compared to unfractured SP KO Control mice, but the IL-6 increase the SP KO FX mice was considerable reduced compared to the IL-6 levels in the WT FX mice. A similar pattern was observed in the RAMP1 KO mice for TNFα (**E**), IL-1β (**F**), or NGF (**H**), except the increase in IL-6 (**G**) levels in the RAMP1 KO FX mice was even greater than the increase observed in the WT FX mice. Data are expressed as mean values (pg/mg protein) ± SE (n = 8 per group). *P < 0.05, **P < 0.001, and ***P < 0.0001 *vs* the respective unfractured Control mice; #P < 0.05, ##P < 0.001, and ###P < 0.0001 *vs* WT FX mice.

The effects of fracture on hindpaw skin cytokine and NGF levels were measured in unfractured and fractured WT, Tac1^−/−^, and RAMP1^−/−^ mice (n = 8 per group). Hindpaw skin was collected the day after cast removal (3 weeks post-fracture) and TNFα, IL-1β, IL-6, and NGF protein levels were determined. Figure 4 illustrates that there were no significant differences in basal TNFα, IL-1β, IL-6, and NGF levels in WT, Tac1^−/−^, and RAMP1^−/−^ unfractured control mice. Fracture caused a dramatic increase in cytokine and NGF levels in WT mice compared to unfractured WT controls. The SP deficient mice had no significant changes in hindpaw TNFα, IL-1β, and NGF levels after fracture, and the post-fracture increase in IL-6 was significantly less than the increase observed after fracture in the WT mice (Figure 4A-D). A similar pattern was seen in RAMP^−/−^ fracture mice, with no significant increase in hindpaw TNFα, IL-1β, and NGF levels after fracture, and the post-fracture increase in IL6 was slightly greater than the increase observed after fracture in the WT mice (Figure 4E-H). These results indicate that SP and CGRP signaling mediate the post-fracture up-regulation of TNFα, IL-1β, and NGF expression, but not the increase observed in IL-6 expression after fracture.

### Cytokine induction in hindpaw skin keratinocytes after tibia fracture

**Figure 5:**
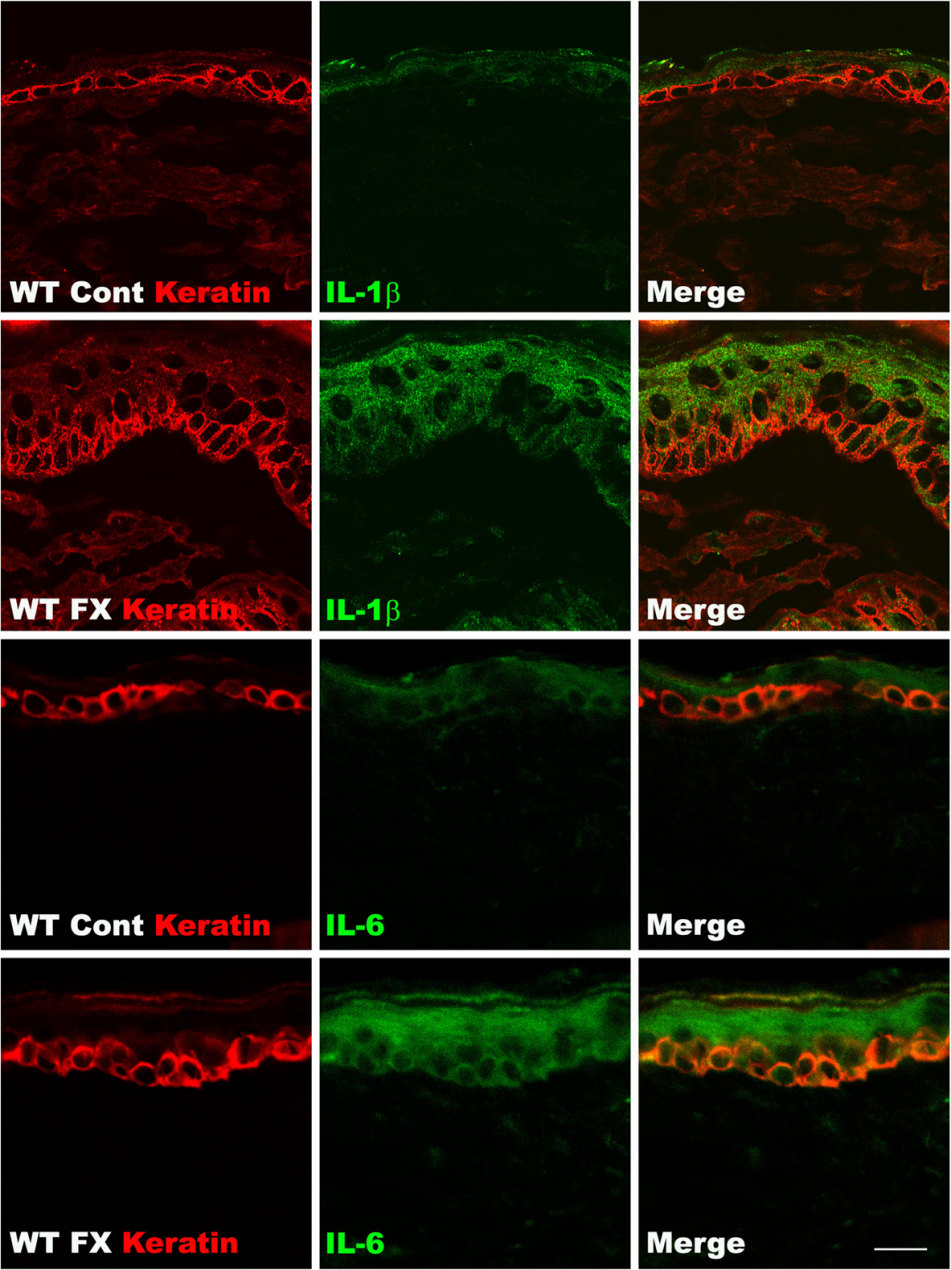
**Fluorescence photomicrographs of keratin (red), IL-1β (green), and IL-6 (green) immunostaining in the plantar hindpaw skin at 3 weeks post-fracture (FX).** The top two rows of panels show keratinocyte and IL-1β immunostaining from a wildtype (WT) unfractured control (Cont) mouse (first row) and the second row panels are from a WT fracture mouse. The bottom two rows of panels show keratinocyte and IL-6 immunostaining from a WT unfractured control mouse (third row) and the bottom row panels are from a WT fracture mouse. Double labeling demonstrates fracture-induced IL-1β and IL-6 protein up-regulation localized within the epidermal keratinocyte layer of the WT mice. Scale bar = 20 μm.

In this study we used confocal microscopy to determine the cellular origin of the IL-1β and IL-6 cytokines in the hindpaw skin after tibia fracture in the mouse. Skin sections were incubated with an antibody directed against keratin (a keratinocyte marker) and co-stained with antibodies against IL-1β or IL-6. Figure 5 shows that hindpaw epidermal keratinocytes in WT control mice expressed a low level of IL-1β and IL-6. In contrast, robust IL-1β and IL6 staining was observed in keratinocytes throughout the epidermis in WT fracture mouse hindpaw skin at 3 weeks post-fracture. These results indicate that keratinocytes are the primary cellular source of the up-regulated IL-1β and IL-6 cytokines observed in hindpaw skin after fracture.

### An IL-6 receptor antagonist reduced post-fracture pain behavior in WT mice

**Figure 6:**
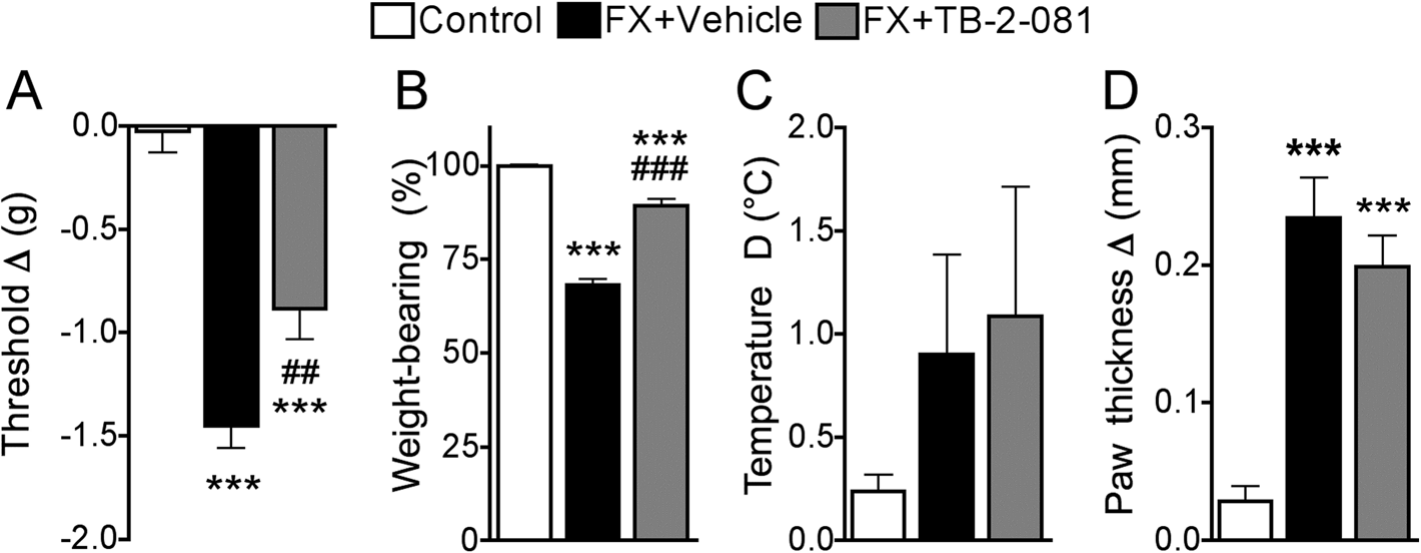
**After baseline testing, WT mice underwent a right distal tibia fracture (FX) and the hindlimb was casted for 3 weeks, then the cast was removed and the next day the animals retested (n = 12 per cohort).** Then the FX mice were either subcutaneously injected with the IL-6 receptor antagonist TB-2-081 (FX + TB-2-081, n = 8) or vehicle (FX + Vehicle, n = 6). A control cohort of WT mice did not undergo tibia fracture (Control, n = 12). At 15 minutes post-injection the mice were retested. The IL-6 receptor antagonist partially inhibited the development of von Frey allodynia (**A**) and hindlimb unweighting (**B**) in the fracture mice, when compared to the extent of allodynia and unweighting observed in the vehicle injected mice after fracture. The IL-6 receptor antagonist failed to reverse the hindpaw warmth (**C**) and edema (**D**) that was observed in the vehicle injected fracture mice. ***P < 0.001 *vs* Control, ##P < 0.01, and ###P < 0.001 *vs* FX + Vehicle.

When the IL-6 receptor antagonist TB-2-081 (2 mg/kg, s.c.) was administered to WT fracture mice there was a partial reduction in hindpaw allodynia and unweighting, but no effect on hindpaw warmth or edema (Figure 6). A lower dose of TB-2-081 (0.2 mg/kg, s.c.) had no effect on allodynia or unweighting in the WT fracture mice (data not shown). These results suggest that the increased cutaneous IL-6 levels observed in the Tac1^−/−^ and RAMP1^−/−^ fracture mice could be responsible for the persistent von Frey allodynia and hindlimb unweighting observed in the transgenic fracture mice.

### Tibia fracture effects on trabecular and cortical bone microarchitecture

**Figure 7:**
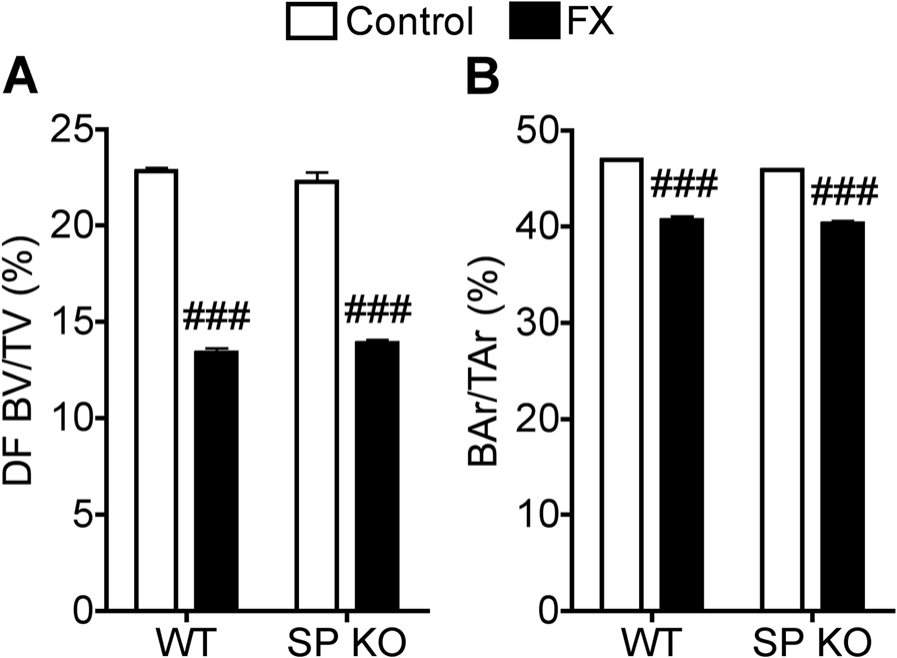
**At 3 weeks after tibia fracture the wildtype (WT) and substance P deficient (SP KO, Tac**^**−/−**^**) mice were euthanized and the femurs ipsilateral to the fracture were collected for uCT evaluation.** No difference was observed in distal femur trabecular bone volumes of WT and SP KO unfractured control mice (**A**), but there was a dramatic post-fracture reduction in distal femur bone volume in both strains of mice relative to their respective unfractured controls (n = 10 per cohort). There was no difference in the magnitude of post-fracture trabecular bone loss between WT and SP deficient mice. Fracture also caused a slight reduction in midfemur cortical bone area in both the WT and SP KO mice (**B**). There were no differences between the WT and SP KO in either baseline or post-fracture midfemur relative cortical areas. ###P < 0.001 *vs* respective unfractured controls.

MicroCT scanning was used to evaluate microarchitecture in the bilateral femurs obtained from WT and Tac1^−/−^ mice at 3 weeks after tibia fracture (n = 10 per cohort). The distal femur trabecular structural parameters evaluated included bone volume fraction (BV/TV, %), trabecular number (Tb.N, mm^−1^), trabecular thickness (Tb.Th, μm), trabecular separation (Tb.Sp, μm), and connectivity density (ConnD, mm^−3^). Cortical mid-femur bone evaluation included the total cross-sectional area (TtAr, mm^2^), cortical bone area (BAr, mm^2^), and the bone area fraction (BAr/TtAr, %). There were no differences in the trabecular or cortical bone parameters of the unfractured WT and Tac1^−/−^ mice (Figure 7, Table 1). Tibia fracture caused a 41% loss of distal femur trabecular bone volume ipsilateral to the fracture in wildtype mice. Similarly, there was a 38% loss of distal femur bone ipsilateral in the SP deficient mice (Figure 7A). Post-fracture cortical bone area in the midfemur was also reduced to an equal extent in the WT and Tac1^−/−^ mice (Figure 7B). Similarly, there were no significant differences between WT and Tac1^−/−^ mice for fracture effects on any other trabecular and cortical structural parameters evaluated in this study (Table 1).

**Table 1:** μCT skeletal morphology of the ipsilateral femurs in the control and tibia fractured (FX) wild type and SP deficient mice at 3 weeks post-injury

	***Wildtype mice***			***SP deficient mice***		
	***Control***	***FX***	***MPD***	***Control***	***FX***	***MPD***
*Distal femur*						
BV/TV (%)	22.8 ± 0.7	13.4 ± 0.8	−41.2%***	22.3 ± 1.3	13.9 ± 0.5	−37.7%***
TbN (mm^-1^)	5.1 ± 0.1	4.6 ± 0.1	**−9.8**%***	5.3 ± 0.1	4.7 ± 0.1	−11.3%***
TbTh (μm)	58.5 ± 0.5	47.6 ± 0.7	−18.6%***	54.7 ± 1.6	46.6 ± 0.5	−14.8%***
TbSp (μm)	185 ± 3	209 ± 5	13.0%***	177 ± 4	203 ± 3	14.7%***
ConnD (1/mm^3^)	156 ± 7	106 ± 8	−32.1%***	181 ± 6	125 ± 5	−30.9%***
*Middle femur*						
BAr (mm^2^)	1.04 ± 0.01	0.90 ± 0.02	13.5%***	0.97 ± 0.04	0.85 ± 0.02	12.4%*
TtAr (mm^2^)	2.22 ± 0.04	2.22 ± 0.06	0	2.11 ± 0.07	2.12 ± 0.05	0.5%
MeAr (mm^2^)	1.18 ± 0.03	1.32 ± 0.06	11.9%*	1.14 ± 0.04	1.27 ± 0.04	11.4%*
CtTh (μm)	224 ± 2	189 ± 5	−15.6%***	211 ± 5	185 ± 4	−12.3%***
BPm (mm)	9.9 ± 0.1	10.3 ± 0.2	4.0%	9.8 ± 0.2	10.0 ± 0.1	2.0%
BAr/TtAr (%)	47.0 ± 0.4	40.7 ± 1.1	13.4%***	45.9 ± 0.4	40.3 ± 0.8	12.2%***

The changes of trabecular and cortical bone parameters in wild type and SP-deficient mice are expressed as the Mean Percent Difference (MPD). MPD = ((FX - Control)/Control) × 100. Bold font indicates statistically significant differences in MPD between FX and age-matched controls for both wild type and SP deficiency mice, as determined by Students’ *t*-test (mean ± SE, *p < 0.05, **p < 0.01 and ***p < 0.001).

## Discussion

In this study we describe a novel mouse fracture model that resembles CRPS in; 1) traumatic etiology and natural history, 2) vascular changes, 3) mechanical sensitivity and guarding, 4) up-regulation of cutaneous inflammatory mediators, and 5) regional periarticular bone loss. After a tibia fracture and 3 weeks cast immobilization the WT mice developed chronic allodynia, unweighting, warmth, and edema (Figure 2), and these vascular and nociceptive changes gradually resolved over a period of 16 weeks (Figure 3).

In an earlier investigation we tested the hypothesis that exaggerated neurogenic inflammatory responses contribute to the vascular and nociceptive sequelae of fracture in rats. When fracture rats were treated with a single-dose of an SP NK1 receptor antagonist there was a 51% reduction in hindpaw spontaneous protein extravasation, a 56% decrease in edema, and a 40% reduction in von Frey fiber allodynia [9]. The results of the current study confirm and extend these findings. SP deficient (Tac1^−/−^) fracture mice had 54% less von Frey allodynia, 70% less hindpaw unweighting, and no hindpaw warmth or edema, compared to WT fracture mice at 3 weeks post-injury (Figure 2). Time-course studies demonstrated further differences in the magnitude and duration of post-fracture nociceptive behaviors in the WT and Tac1^−/−^ mice. The WT mice developed nociceptive behaviors persisting for 8–15 weeks after fracture, while in the SP deficient mice nociceptive behaviors persisted for only 4–6 weeks post-fracture (Figure 3). These data confirm that SP is a critical regulator for the development of nociceptive and vascular sequelae after fracture. Similarly, CGRP receptor deficient (RAMP1^−/−^) fracture mice had attenuated allodynia and hindpaw unweighting, and no hindpaw warmth, but post-fracture edema was not affected by the loss of CGRP signaling (Figure 2). The contribution of SP and CGRP signaling to post-fracture hindpaw warmth and the essential role of SP signaling in the development of post-fracture hindpaw edema correlates with the results of intradermal microdialysis studies in human subjects demonstrating that SP or CGRP perfusion induces a vasodilation response in the skin, but only SP perfusion can evoke a protein extravasation response [30]. In addition, the sciatic nerve electrically evoked protein extravasation response was dramatically reduced in Tac1^−/−^ mice and unchanged in the RAMP1^−/−^ mice (Figure 1C), indicating that CGRP signaling does not contribute to neurogenic extravasation in mice. Spontaneous protein extravasation is up-regulated in CRPS affected limbs [31], leading to increased interstitial fluid flow and distal limb edema, an important component of CRPS [12].

When SP or CGRP is microdialyzed through the skin of normal volunteers [30] or in CRPS skin [18] there is no immediate painful response, leading us to postulate that SP and CGRP act as intermediate mediators in the development of post-traumatic pain. Increased levels of the inflammatory cytokines TNFα and IL-6 are observed in experimental blister fluid or skin biopsies from the affected, but not the contralateral limbs of CRPS patients [23-25]. TNFα, IL-1b, IL-6, and NGF are up-regulated in the hindpaw skin at 4 weeks after tibia fracture in rats and fracture rats treated systemically with the TNF inhibitor etanercept, the IL-1 receptor antagonist anakinra, or the NGF antibody tanezumab had reduced allodynia and hindlimb unweighting, indicating an important role for cytokine and growth factor signaling in the development of trauma induced chronic pain [26,27,29].

The TNF, IL-1, and NGF inhibitors we have tested in the fracture rat model are large molecules that can’t cross the blood-brain barrier, suggesting that the pronociceptive effects of these inflammatory mediators probably occur at the nociceptor level in the sensitized hindpaw skin. Pro-inflammatory cytokines and NGF can immediately evoke spontaneous firing and sensitization in primary sensory afferents [32-34]. Furthermore, we have observed that intraplantar injection of these inflammatory mediators into normal hindpaw skin rapidly induces nociceptive sensitization and have identified keratinocytes as the primary cellular source of these inflammatory mediators in the fracture hindpaw (Figure 5) [28]. Robust keratinocyte proliferation and epidermal thickening was also observed in the fracture hindpaw [28]. Collectively, these data indicate that up-regulated keratinocyte proliferation and/or inflammatory mediator expression results in increased cutaneous inflammatory mediator levels in the fracture hindpaw, with subsequent nociceptive sensitization.

Neurotransmitter release evoked by capsaicin injection or electrical nerve stimulation stimulates the production of TNFα, IL-1β, IL-6, and NGF in the skin of rats, but it is unknown which specific transmitters mediate these inflammatory effects [35,36]. Previous studies have observed that SP and CGRP signaling can stimulate the secretion of IL-1β and NGF from keratinocyte cell cultures, *in vitro* evidence that these neuropeptides can directly stimulate keratinocyte inflammatory mediator release [37-39].

Figure 4 illustrates the robust increase in TNFα, IL-1β, IL-6, and NGF protein levels observed in the hindpaw skin at 3 weeks post-fracture in the WT mice. Cytokines are normally expressed transiently in response to injury and infection and the persistent regional elevation of cytokines observed at 3 weeks post-fracture indicates ongoing cytokine synthesis by the keratinocytes (Figure 5), which we postulate is the result of exaggerated neuropeptide signaling. In support of this hypothesis, Tac1 or RAMP1 gene deletion prevented the up-regulation of TNFα, IL-1β, and NGF in the injured hindlimb (Figure 4). There were no differences between the WT, Tac1^−/−^ and RAMP1^−/−^ mice in baseline cutaneous cytokine and NGF levels, evidence that the low basal levels of cytokines and NGF expressed in normal skin are not dependent on neuropeptide signal.

Both the Tac1^−/−^ and RAMP1^−/−^ fracture mice had increased IL-6 levels in the hindpaw skin, and there was residual, albeit attenuated, allodynia and unweighting in these transgenic fracture mice compared to WT fracture mice (Figures 2,4). We postulated that IL-6 signaling contributes to post-fracture allodynia and unweighting and that the increase in skin IL-6 levels after fracture in the Tac^−/−^ and RAMP1^−/−^ mice is the mechanism for the residual post-fracture allodynia and hindpaw unweighting observed in these strains. To test this hypothesis we treated WT fracture mice with an IL-6 receptor antagonist (TB-2-081), and this treatment partially reversed fracture-induced allodynia and unweighting (Figure 6), supporting the hypothesis that increased cutaneous IL-6 signal contributes to allodynia and unweighting after fracture.

This study provides the first in vivo evidence that SP and CGRP act as intermediate mediators in the post-traumatic inflammatory cascade by chronically up-regulating inflammatory proteins that can directly sensitize cutaneous nociceptors. In contrast to other inflammatory mediators examined in this study, the post-fracture up-regulation of IL-6 did not require CGRP signaling and was only partially dependent on SP signaling. The significance of IL-6 signaling was confirmed by antagonist studies demonstrating its role in the maintenance of post-fracture nociceptive behavior. Collectively, these data suggest that neuronal regulation of innate immunity can mediate inflammatory changes observed in CRPS skin.

After tibia fracture WT mice developed trabecular, and to a lesser extent cortical bone loss in the ipsilateral femur when compared to unfractured controls (Figure 7, Table 1). These data are consistent with the results observed in the rat tibia fracture model [9], as well as in CRPS patients who develop periarticular bone loss after fracture [40]. The net effects of SP signaling on bone acquisition and resorption in vivo are unknown, but previously we observed that the SP NK1 receptor is expressed in both bone marrow stromal cells and bone marrow macrophages and SP signaling stimulates bone marrow stromal cell osteogenic activity as well as macrophage osteoclast differentiation and resorption activity *in vitro*[41]. The WT and SP deficient (Tac1^−/−^) mice had similar baseline bone parameters and no post-fracture differences were observed in skeletal integrity between groups, indicating that SP signaling does not regulate post-traumatic bone loss in mice (Figure 7 and Table 1).

CRPS patients usually have deep tissue pain, primarily tenderness over the distal limb joints and pain with range of motion [15,42]. It is unknown whether the neuroinflammatory changes observed in the fracture limb skin may also be occurring in the joints or muscles of the injured limb, but there are some intriguing clues. The most prominent histological finding in CRPS synovial biopsies is the proliferation of synovial lining cells [15]. Synoviocytes express NK1 receptors and very low concentrations of SP can stimulate synoviocyte proliferation *in vitro*[43,44]. Stimulated synoviocytes express IL-6 and CCL2, a chemokine which induces monocytes to leave the bloodstream and enter the surrounding tissue to become synovial macrophages, the primary source of TNFα and IL-1β in rheumatoid joints [45]. Examining neuroinflammatory articular changes after fracture could potentially identify signaling mechanisms contributing to the articular tenderness and joint motion pain associated with CRPS.

## Conclusions

There have been numerous unsuccessful clinical trials testing the analgesic efficacy of NK1 antagonists in post-herpetic pain, diabetic neuropathy, osteoarthritis, and migraine patients [46]. Only post-operative pain has been responsive to NK1 antagonist analgesia in clinical trials [47] and our previous work with the hindpaw incision post-operative pain model in SP deficient mice [48] suggests that SP signaling contributes to both the post-incision increase in inflammatory mediator levels in the traumatized skin and the subsequent post-operative pain. We postulate that the mixed results of the NK1 antagonist clinical trial data reflect differing pathophysiologic mechanisms underlying the various pain etiologies examined in these trials. CGRP receptor antagonists have been demonstrated to effectively reduce pain in migraine patients but have not been tested in other types of pain patients [49]. The results of the current study supports the hypothesis that SP and CGRP signaling contribute to the post-fracture up-regulation of cutaneous inflammatory mediators capable of inducing pain behaviors in the mouse CRPS model and these data provide a translational basis for future clinical trials with NK1 or CGRP antagonists in early CRPS.

## Methods

### Study design

Baseline determinations were made of bilateral hindpaw von Frey withdrawal thresholds, weight bearing, temperature, and thickness in the wildtype (WT), SP deficient (Tac1^−/−^), and CGRP receptor deficient (RAMP1^−/−^) mice. Then the mice underwent a right distal tibia fracture with cast immobilization for 3 weeks, then the cast was removed and the mice were retested the following day. One group of WT mice did not undergo fracture and served as controls. Two groups of additional groups of control mice were used in these experiments; 1) WT mice that had a crush injury (no fracture or cast) to the distal tibia using the same hemostat used to fracture the tibia, and 2) WT mice that underwent hindlimb cast immobilization for 3 weeks with no fracture. After behavioral testing the mice were euthanized and the bilateral hindpaw skin and femurs were collected. Another group of WT fracture mice underwent behavioral testing the day after cast removal and the next day were subcutaneously injected with either the IL-6 receptor antagonist TB-2-081 [50] or vehicle (50% PBS/50%EtoH, total volume 50 ul) and 15 min later behavioral testing was repeated. Another group of WT and Tac1^−/−^ fracture mice underwent long-term behavioral testing over an 18-week period after cast removal. Electrically evoked protein extravasation responses were evaluated in unfractured WT, Tac1^−/−^, and RAMP1^−/−^ control mice to confirm our hypothesis that the SP deficient mice had impaired neurogenic extravasation and that RAMP^−/−^ mice had normal extravasation responses.

### Mice

Mice that were homozygous for a disruption of the SP expressing Tac1 (Tac1^−/−^) gene (B6.Cg-Tac1^tm1Bbm^/J) and wildtype (WT) controls (C57Bl/6) were obtained from Jackson Laboratory. CGRP receptor binding requires the receptor activity-modifying protein 1 (RAMP1). Mice that were homozygous for disruption of the RAMP1 gene (RAMP1^−/−^) were generated by Dr Kazutake Tsujikawa (Dept Immunology, Osaka University) on a C57Bl/6 background [51]. The Cre-loxP system was used to delete exon 2 of the RAMP1 gene, with splicing of exons 1 to 3 creating a frameshift resulting in a termination codon at the beginning of exon 3.

The genotype of each breeding pair was confirmed by extracting DNA from tail snips and performing a PCR assay using an Extract-N-AmpTM kit (XNAT-1KT, Sigma). Forward (oIMR2033, 5′-GCT CAT CAG TAT GTG ACA TAG AAA) and reverse (oIMR2032, 5′-AGA ATT TAA AGC TCT TTT GCC) primers were used to identify the Tac1^−/−^ mice, based on the Jackson Laboratory’s genotyping protocol. The Tac1^−/−^ mice yield shorter PCR product (size 130 bp) on a 3% agrose gel, whereas WT mice yielded longer product (180 bp). Genotyping for the RAMP1^−/−^ mice used a 5′-specific primer upstream of the 5′ site in intron 1 (5′-CAGAATGGAGAAACTGAGTAGAGC-3′), a 3′-specific primer immediately downstream of the first loxP site in intron 1 (5^′^-AGGAAGGAACGTAACACAGGTGG-3^′^), and a 3′-specific primer downstream to the last loxP site (5^′^-GCTGTGCGTGGTGATGGAGG-3′). The PCR reaction conditions were: 40 cycles of a reaction consisting of 30 s of denaturation at 94°C, 30 s of annealing at 59°C, and 60 s of elongation at 72°C [51]. Wildtype and RAMP1^−/−^ mice yielded PCR products of 108 and 240 bp, respectively.

The Tac1^−/−^ mice are viable, fertile, normal in size and do not display any gross physical or behavioral abnormalities [52]. SP is not immunodetectable in the Tac1^−/−^ mice and these mice have slightly reduced nociceptive responses to moderate/intense pain stimuli [52]. The RAMP1^−/−^ mice are also viable, fertile, normal in size, and do no display any gross physical abnormalities [51]. The RAMP1^−/−^ mice have hypertension without tachycardia, exhibit no vasodilation response to intravenous CGRP, and are incapable of nurturing their new born pups, thus requiring surrogate mothers to raise their litters [51]. All experiments were done with the approval of our Institutional Animal Care and Use Committee.

### Surgery

The fracture model was performed in 3 month-old male mice. Under isoflurane anesthesia a hemostat was used to make a closed fracture of the right tibia just distal to the middle of the tibia. Then the hindlimb was wrapped in casting tape (Delta-Lite) so the hip, knee and ankle were all fixed. The cast extended from the metatarsals of the hindpaw up to a spica formed around the abdomen. A window was left open over the dorsal paw and ankle to prevent constriction when post-fracture edema developed. After fracture and casting, the mice were given subcutaneously 2 days of Buprenorphine (0.05 mg/kg) and Baytril (5 mg/kg) as well as 1.5 ml of normal saline. At 3 weeks after surgery the mice were anesthetized with isoflurane and the cast removed. All mice had union at the fracture site by manual inspection.

### Hindpaw hyperalgesia and unweighting

Mechanical allodynia was assayed using nylon von Frey filaments according to the “up-down” algorithm as previously described [53]. The mice were placed on wire mesh platforms in clear cylindrical plastic enclosures 10 cm in diameter and 40 cm in height, and after 15 minutes of acclimation von Frey fibers of sequentially increasing stiffness were applied against the hindpaw plantar skin at approximately midsole, taking care to avoid the tori pads, and pressed upward to cause a slight bend in the fiber and left in place for 5 sec. Withdrawal of or licking the hindpaw after fiber application was scored as a response. When no response was obtained the next stiffest fiber in the series was applied to the same paw; if a response was obtained a less stiff fiber was applied. Testing proceeded in this manner until 4 fibers had been applied after “negative + positive or positive + negative” response. Estimation of the mechanical withdrawal threshold by data fitting algorithm permitted the use of parametric statistics for analysis [54]. Baseline von Frey withdrawal thresholds were identical in the WT, Tac1^−/−^, and the RAMP1^−/−^ mice.

An incapacitance device (IITC Inc) was used to measure hindpaw unweighting. The mice were manually held in a vertical position over the apparatus with the hindpaws resting on separate metal scale plates and the entire weight of the mouse was supported on the hindpaws. The duration of each measurement was 6 s and 6 consecutive measurements were taken at 10 s intervals. All 6 readings were averaged to calculate the bilateral hindpaw weight-bearing values.

### Hindpaw volume

A laser sensor technique was used to determine the dorsal–ventral thickness of the hindpaw, as we have previously described [29]. The measurement sensor device used in these experiments (Limab) has a measurement range of 200 mm with a 0.01 mm resolution.

### Hindpaw temperature

The temperature of the hindpaw was measured using a fine wire thermocouple (Omega) applied to the paw skin, as previously described previously [29]. The investigator held the wire using an insulating Styrofoam block. Three sites were tested over the dorsum of the hindpaw: the space between the first and second metatarsals (medial), the second and third metatarsals (central), and the fourth and fifth metatarsals (lateral). After a site was tested in one hindpaw the same site was immediately tested in the contralateral hindpaw. The testing protocol was medial dorsum right then left, central dorsum right then left, lateral dorsum right then left, medial dorsum left then right, central dorsum left then right, and lateral dorsum left then right. The six measurements for each hindpaw were averaged for the mean temperature.

### Enzyme immunoassay for TNFα, IL-1β, IL-6 and NGF levels in hindpaw skin

The mice were deeply anesthetized and bilateral dorsal and plantar hindpaw skin were collected rapidly and placed into ice cold 0.9% NaCl containing a cocktail of protease inhibitors (Complete™, Roche Applied Science), homogenized, then centrifuged (10 min, 12,000 G, 4°C), then supernatant fractions were frozen at −80°C. An aliquot was subjected to protein assay (DC Protein Assay, Bio-Rad) to normalize mediator levels. Cytokine protein levels were determined using a custom Bio-Plex kit for TNFα, IL-1β, and IL-6, analyzed on a Bio-Plex system array reader (Bio-Rad) as previously described [55]. Samples were diluted 1:2 prior to analysis in the buffer supplied, and all samples were run in duplicate for each assay. We demonstrated previously that the dynamic range of sensitivity of this assay was sufficient to measure both baseline and incision-stimulated levels of the chosen cytokines [55]. Standard curves for each of the analyzed substances were included in each run, and sample concentrations were calculated using Bio-Plex Manager software. NGF levels were determined using the ChemiKine EIA kit (Chemicon). Sample preparation was identical to that described for the cytokines.

### Western blot assay for RAMP1 protein levels in hindpaw skin

Western blotting was carried out to confirm RAMP1 protein knockout in the RAMP1^−/−^ mice. Hindpaw skin of WT and RAMP1^−/−^ mice was collected under isoflurane anesthesia and homogenized in modified RIPA buffer (50 mM Tris–HCl, 150 mM NaCl, 1 mMEDTA, 1% Igepal CA-630, 0.1% SDS, 50 mM NaF, and 1 mM NaVO3) containing protease inhibitors (aprotinin (2 μg/ml), leupeptin (5 μg/ml), pepstatin (0.7 μg/ml), and PMSF (2 mM); Sigma). The homogenate was centrifuged at 13,000 g for 30 min at 4°C. Total protein concentration of the homogenate was measured using a Coomassie Blue Protein Assay (Bio-Rad) and BSA protein standard (Pierce). The supernatant was subjected to western blot analysis. Equal amounts of protein (50 μg) were subjected to SDS-PAGE (12% Tris–HCl acrylamide gel, Bio-Rad) and electrotransferred onto a polyvinylidene difluorided membrane (Millipore). The blots were blocked with 5% non-fat dry milk in tris-buffered saline, incubated with goat anti-RAMP1 antibody (Novus Biologicals) overnight at 4°C and further incubated with HRP-conjugated secondary antibody (Santa Cruz Biotechnology) for 1 hr at room temperature. After washing in TBST three times, the blot was then incubated in ECL plus chemoluminescence reagents (Amersham) and scanned by PhosphoImager (Typhoon, GE Healthcare) to detect specific bands.

### Immunofluorescence confocal microscopy

The mice were euthanized and perfused with 50 ml 4% paraformaldehyde (PFA) in phosphate buffered saline, pH 7.4, via the ascending aorta; the plantar hindpaw skin including sub-dermal layers was removed and post-fixed in 4% PFA for 2 hours, then the tissues were treated with 30% sucrose in PBS at 4°C before embedding in OCT. Following embedding, 8-μm thick slices were made using a cryostat, mounted onto Superfrost microscope slides (Fisher Scientific), and stored at −80°C. Frozen sections were permeabilized and blocked with PBS containing 10% donkey serum and 0.3% Triton X-100, followed by exposure to the primary antibodies overnight at 4°C in PBS containing 2% serum. Upon detection of the first antigen, primary antibody from a different species against the second antigen was applied to the sections and visualized using an alternative fluorophore-conjugated secondary antibody. Sections were then rinsed in PBS and incubated with fluorophore-conjugated secondary antibodies against the immunoglobulin of the species from which the primary antibody was generated. After three washes, the sections were mounted with anti-fade mounting medium (Invitrogen). Images were obtained using a confocal microscope (Zeiss LSM/510 Upright 2 photon; Carl Zeiss) and stored on digital media.

For IL-6 immunostaining rabbit anti human IL-6 antibody (1:400, LifeSpan Biosciences Inc) was labeled with donkey anti-rabbit IgG (1:500) conjugated with Dylight 488 secondary antibodies (Jackson ImmunoResearch). Control experiments included incubation of slices in primary and secondary antibody-free solutions both of which led to low intensity non-specific staining patterns in preliminary experiments. For the detection of epidermal keratinocytes, rabbit anti keratin 14 polyclonal antibody (Convance Inc) was labeled with Alexa Fluor 555 microscale protein labeling kit (Invitrogen Molecular Probes) under optimum conditions according to the manufacture’s protocol, and quantified using a NanoDrop ND-1000 UV–vis spectrophotometer (NanoDrop Technologies), the concentration of purified protein conjugate was 0.34 mg/ml. Then an equal volume of glycerol (ACS grade) was added for a final concentration of 50%. Upon detection of the IL-6 as described above, 250 μl of a 1:200 dilution of the conjugate was used for the keratin 14 immunostaining. Control experiments included incubation of slices in primary and secondary antibody-free solutions both of which led to low intensity non-specific staining patterns in preliminary experiments.

For IL1-b immunostaining, the primary antibodies, rabbit anti-mouse IL-1b, 1:200 (Millipore) and mouse monoclonal antibody to Pan Keratin, 1:50 (Abcam) were used. Double labeling immunofluorescence was performed with donkey anti-mouse IgG (1:500) conjugated with Dylight 549, donkey anti-rabbit IgG (1:500) conjugated with Dylight 488 secondary antibodies (Jackson ImmunoResearch), incubated with respective primary antibodies. Control experiments included incubation of slices in primary and secondary antibody-free solutions both of which led to low intensity non-specific staining patterns in preliminary experiments.

### Electrical stimulation-evoked extravasation procedure

Under isoflurane anesthesia both sciatic nerves were exposed in the thigh and tightly ligated proximal to the midthigh stimulation site. Then the incision was filled with warm mineral oil and a Plexiglas-platinum stimulating electrode was gently secured around the nerve (Harvard Apparatus). Evans blue dye (25 mg/kg, Sigma) dissolved in saline was administered through the jugular vein, and 10 minutes later bilateral sciatic nerves were stimulated for 5 min (5 Hz, 0.5-ms pulse duration, 10 mA) and then the mice were transcardially perfused with 30 ml normal saline. The plantar and dorsal skin on each hindpaw was excised and then placed in 1 ml of formamide in a shaker bath at 55°C for 72 h. The extracted dye concentration was determined with a spectrophotometer at 620 nm. Because Evans blue binds to serum albumin, the dye content of the hindpaw skin provides an accurate measure of electrically evoked protein extravasation into the interstitial space.

### Microcomputed tomography (μCT)

To assess trabecular and cortical bone architecture, *ex vivo* scanning was performed using μCT (VivaCT 40, Scanco Medical AG) as we have previously described [27]. In the distal femur, 160 transverse slices of 10.5 μm thickness (10.5-μm isotropic voxel size) encompassing a length of 1.68 mm were acquired, but only 100 slices encompassing 1 mm in the distal femur were evaluated, starting where the growth plate bridge across the middle of the metaphysis ends. The region of interest (ROI) was manually outlined on each CT slice, excluding the primary spongiosa, and extending distally from the growth plate. Image threshold was set by using an adaptive-iterative algorithm for trabecular bone. Scan parameters of threshold = 252, sigma = 0.8, support = 1 for proximal tibia were used to measure trabecular bone volume fraction (BV/TV, %), trabecular number (Tb.N, mm^−1^), trabecular thickness (Tb.Th, μm), trabecular separation (Tb.Sp, μm), and connectivity density (ConnD, mm^−3^) [56,57].

For the mid-femur cortical bone evaluation, a total of 26 transverse CT slices were obtained at the mid-femur, each 21 μm thick totaling 0.546 mm in length (21 μm isotropic voxel size). Scan parameters of threshold = 350, sigma = 1.2, and support = 1 were used to measure total cross-sectional area (Tt.Ar, mm^2^), cortical bone area (B.Ar, mm^2^), cortical thickness (Ct.Th, μm), medullary area (Me.Ar, mm^2^), and bone perimeter (B.Pm, mm). Relative cortical bone area (B.Ar/T.Ar, %) was calculated.

### Statistical analysis

A two-tailed Student’s *t*-test was used to analyze differences between groups. A two-way repeated measures ANOVA was used to analyze nociceptive and vascular behavioral data for differences between groups over time. All data were presented as the mean ± standard error of the mean, and differences were considered significant at a P value less than 0.05. Hindpaw temperature, thickness, and mechanical nociceptive thresholds data were analyzed as the difference between the fracture side (right) and the contralateral (left) side. Hindpaw weight bearing data was analyzed as a ratio between the right hindpaw weight and the sum of right and left hindpaws values ((2R/(R + L)) × 100%).

## Competing interests

The authors declare that they have no competing interests.

## Authors’ contributions

TG generated the fracture mice, performed the behavioral experiments, analyzed the data, generated figures, and contributed to the writing of the manuscript. TW performed some of the biochemical assays, analyzed the data, generated figures, and contributed to the writing of the manuscript. XS performed most of the biochemical assays, analyzed the data, and generated figures. WL performed the immunohistochemistry, analyzed the data, and generated figures. SH and LW bred the transgenic mice and genotyped them. KT generated the RAMP1^−/−^ mice. KR and KC generated the TB-2-081 used in this study. DC participated in the design, data analysis, and editing of this manuscript. WK participated in the design, data analysis, and editing of this manuscript. All the authors have read and approved the final manuscript.
